# Genome-Wide Identification of *GRAS* Gene Family in Daylily (*Hemerocallis citrina* Baroni) and Its Expression Profiles in Development, Hormone and Biotic Stress Response

**DOI:** 10.3390/biology14070770

**Published:** 2025-06-26

**Authors:** Weijia Li, Hongying Sun, Zhiwen Chen, Yue Zhang, Jianguo Zhao

**Affiliations:** 1 Engineering Research Center of Coal-Based Ecological Carbon Sequestration Technology of the Ministry of Education, Shanxi Datong University, Datong 037009, China; 2 Department of Horticulture, Shenyang Agricultural University, Shenyang 110866, China; 3 College of Agronomy and Life Science, Shanxi Datong University, Datong 037009, China

**Keywords:** *Hemerocallis citrina* Baroni, *GRAS* gene family, genome-wide analysis, expression pattern

## Abstract

The family of *GRAS* transcription factors plays an essential role in the regulation of plant development, the transmission of hormonal signals, and the adaptation to environmental stresses seen in numerous species. However, a comprehensive analysis of the *GRAS* family of *Hemerocallis citrina* (daylily) is lacking, despite its potential to help understand the stress content and developmental processes of the monospecies. This study identified *78 GRAS* genes (*HcGRAS*) in *H. citrina*, which were classified into 15 subfamilies based on chromosomal location, gene structure, conserved motifs, and expression patterns. An analysis of promoter regions indicated a significant presence of elements related to hormones and stress, showcasing these genes’ role in adapting to environmental pressures. The GO and KEGG analyses indicated that *HcGRAS* genes were engaged in pathways associated with developmental processes and responses to environmental stress. Notably, *HcGRAS38* was identified as a key interacting protein for SHR, SCR, and DELLA subfamily members, which suggested its central role in coordinating stress response and developmental signaling. Comparative genomic mapping with seven representative monocot and dicot species underscored evolutionary conservation and divergence in functions of the *GRAS* family. The expression profiling of 22 *HcGRAS* genes across different daylily tissues provided insights into their tissue-specific roles. This research may promote the further exploration of the functional characteristics of *HcGRAS* genes.

## Simple Summary

This study examines the *GRAS* gene family in daylily, focusing on its role in flower development, hormone response and biotic stress. A total of 78 *HcGRAS* genes were identified and divided into 15 subfamilies. These genes encode basic, hydrophilic, unstable nuclear proteins and are distributed across 11 chromosomes, with segmental duplication driving their expansion. The transcriptome data and RT-PCR indicate that these genes are involved in organ development, hormone response, and rust and thrips response. These findings lay the groundwork for future research on the regulatory mechanisms of *HcGRAS*s in development and response to biotic stress.

## 1. Introduction

The GRAS transcription factors (TFs) represent a significant plant-specific TF family that consists of three members, which are gibberellin acid-insensitive (GAI), repressor of GA1 (RGA) and scarecrow (SCR) [1]. *GRAS* is involved in almost the whole process of plant growth and development, which contributes much to the preservation of shoot meristems, the initiation of lateral meristems, male gametogenesis, root development, gibberellin (GA) and light signal transduction and stress resistance [2,3,4]. All the *GRAS* genes are monoexonic, meaning they encode proteins with a length of 360–850 amino acids [1]. The GRAS protein family is characterized by a relatively conserved C-terminal domain paired with a more diverse N-terminal domain. The C-terminal consists of five extremely conserved fragments: SAW, PFYRE, VHIID, LHRI, and LHRII [4,5]. Between LHRI and LHRII, VHIID serves as a conserved domain in nearly each *GRAS* protein. The LHRI-VHIID-LHRII pattern is crucial to the binding of proteins to nucleic acids or other proteins [6,7,8]. Additionally, SAW and PFYRE motifs are associated with the structural integrity of *GRAS* proteins [8]. Sequences at the N-terminal vary and are involved in distinct signal transduction pathways, which can decide their functions’ specificity [5].

The *GRAS* family exists in a variety of species, such as thale cress (*Arabidopsis thaliana*) [2,9], rice (*Oryza sativa*) [2], soybean (*Glycine max*) [10], foxtail millet (*Setaria italica* L.) [10], sugar beet (*Beta vulgaris*) [11], and six *Rosaceae* species [8]. The classification of the *GRAS* family includes several subfamilies, including DELLA, LS (lateral suppressor), LISCL (*Lilium longiflorum* SCR-like), NSP (nodulation signaling pathway), PAT1 (phytochrome a signal transduction 1), HAM (hairy meristem), SCR (scarecrow), SCL3 (scarecrow-like 3), and SHR (short root) [12,13]. However, a different number of subfamilies were reported [14,15,16,17], whose respective phylogenetic results were defined [18]. *GRAS* proteins exhibit different structures and play multiple roles in the growth and development of plants [19]. For instance, AtSCR participates in the process of cell division in the root cortex and endocortex, while SHR activates SCR proteins to regulate the asymmetric division of roots [14,15,16,17]. *NSP1* and *NSP2* are required for nodulation induced by the rhizobium Nod factor in *Medicago truncatula* [20]. It has been reported that *AtLAS*, *OsMOC1* and *SlyLs* are associated with axillary formation [21,22,23]. HAM family members contribute much to shoot meristem development [24]. The PAT1 subfamily is a well-known branch of the *GRAS* family with multiple regulatory functions in transcriptional regulation and phytochrome signal transduction [25]. DELLA proteins act as crucial negative modulators of GA signaling [26]. *BraRGL1* mutants (a loss-of-function mutation) lead to the rapid differentiation of flower bud and time of bolting [27]. After employing RNAi technology to silence the strawberry *GRAS* gene *FveRGA1*, many runners were produced in naturally non-running YW and RG strains, indicating that *FveRGA1* plays a crucial role in the regulation of runner formation in woodland strawberry [28]. *FveLAM* acts with GA in sequence from bud initiation to runner outgrowth, which cannot be separated from the formation of stamen and runner [29].

Transcription factors, including GRAS, are essential for plant adaptation to biotic and abiotic stresses when responding to environmental signals to regulate growth and development [11,29,30,31,32,33]. Given the current challenges of climate change, including temperature fluctuations and soil variations, the need for stress-resistant crop varieties is more urgent than ever [30]. Open-field agricultural crops are especially vulnerable to these shifting conditions, affecting yields and overall crop resilience. Thus, understanding the function of GRAS genes in stress responses is a significant step in breeding programs aimed at creating high-yielding, resilient crops.

The flower buds of *Hemerocallis citrina* Baroni (daylily) are valuable and widely cultivated in horticulture due to their edibility, and they are extensively enjoyed as a healthy vegetable option across Asia [34]. Daylily is native to China, with its natural distribution spanning regions south of the Qinling Mountains and Hebei, Shanxi, and Shandong provinces. It has also been documented in Japan and Southeast Asia. This species exhibits strong cold resistance, drought tolerance, and adaptation to semi-shaded environments. Its soil requirements are highly adaptable, thriving in diverse conditions such as sandy soils, clay substrates, alluvial plains, and mountainous terrains [34]. The Datong region in Shanxi, one of China’s major production areas, provides unique growth conditions, including selenium-rich soil and extreme diurnal temperature variation, which contribute to the quality and resilience of daylily. The Datong yellow flower planting area belongs to the semi-arid to arid region, with an average annual rainfall of 300–400 mL [35]. A study of GRAS TFs in daylily can provide insights into the genetic foundation of this adaptability and may inform breeding efforts aimed at enhancing stress resistance and yield. Moreover, these findings could have broader applications for other crops grown in similar environments, facilitating an understanding of how GRAS TFs contribute to crop resilience.

This research comprehensively characterized *GRAS* TFs in daylily at the genome-wide level for the first time, including an analysis of gene duplication, conserved structure, phylogeny, and physicochemical properties across different flower development stages, salicylic acid (SA) and jasmonic acid (JA) treatment, and thrips disease and rust treatment. Such findings are likely to not only offer support for future functional explorations of GRAS genes in daylily but also guide breeding efforts to enhance the adaptability and nutritional value of this crop and potentially others.

## 2. Materials and Methods

### 2.1. Plant Materials and Data Resources

The daylily variety in this study was *H. citrina* Baroni cv. Datonghuanghua, which was collected in July 2024 from the Tangjiapu Organic Daylily Standardized Planting Base, Yunzhou District, Datong, Shanxi Province, China (40°2′1″ N, 113°36′4″ E, altitude ≈ 1157 m). The cultivation base for daylily is characterized by a temperate continental semi-arid monsoon climate, with an average annual temperature of 6.4 °C and annual precipitation of 391.5 mm. Its volcanic ash-derived soil is notably enriched with zinc and selenium. The research samples included leaves, roots, stems, and flowers in the developmental stage: F1 (1–2 cm), F3 (3–4 cm), F5 (5–6 cm), F7 (7–8 cm), F9 (9–10 cm) and stage F (full opening). Additional information can be found in Supplementary Figure S1. For each development stage, three samples were collected. For each type of sample, we took random samples from different plants in the daylily planting areas. Before being stored at −80 °C, all samples were flash frozen in liquid nitrogen.

The genome sequences of *A. thaliana* and *H. citrina* Baroni were obtained from TAIR (http://www.arabidopsis.org, accessed on 23 April 2024) and the NCBI (https://www.ncbi.nlm.nih.gov/Traces/wgs/JACEHZ01?val=JACEHZ01, accessed on 23 April 2024) [34]. In addition, those from *O. sativa* (GCF_034140825.1), *Brachypodium distachyon* (GCF_000005505.3), *Sorghum bicolor* (GCA_039639735.1), *S. italica* (GCF_000263155.2), *Solanum lycopersicum* (GCA_025759795.1) and *Zea mays* (GCA_001644905.2) were sourced from Phytozome 13.0 (https://phytozome-next.jgi.doe.gov/, accessed on 23 April 2024). The RNA-seq data from different tissues of daylily (SRX7583022, SRX7583021, SRX7583020, SRX7583019, SRX7583018, SRX7583017, SRX7583016, SRX7583015, and SRX7583014) were systematically retrieved from the NCBI SRA database.

### 2.2. Identification and Characterization of GRAS Genes

This research first retrieved the GRAS genes in species using BLASTP (v2.16.0) at the threshold E-value of 1 × 10^−5^. Then, 56 *Oryza sativa* GRAS proteins (Table S1) were used as query sequences to choose the minimum alignment coverage of 50%. Through the BLASTP analysis, every selected candidate protein was employed to perform a search within the Swissprot database (https://www.uniprot.org/, accessed on 28 May 2024), thus exploring its corresponding ortholog at the specified threshold along with the minimum alignment coverage parameters. These findings were verified by repeating the aforementioned steps. The sequence number of the GRAS genes was obtained through summarizing the aforementioned results. The protein sequences were sourced from the genomic protein dataset of daylily through the utilization of TBtools (v2.310) [36]. The GRAS domain (PF03514) retrieved from Pfam (http://pfam.xfam.org, accessed on 28 May 2024) was employed to identify the potential GRAS genes of *H. citrina* Baroni [37]. To further verify the domain of GRAS, the Conserved Domain Database (CDD) in the NCBI (https://www.ncbi.nlm.nih.gov/Structure/bwrpsb/bwrpsb.cgi, accessed on 28 May 2024) was used [38]. The *HcGRAS* gene structure was manually corrected by employing IGV-GSAman software (version 0.8.4, https://gitee.com/CJchen/IGV-sRNA, accessed on 29 May 2024), with the aid of RNA sequencing data. The updated GFF annotation file was used for further analysis.

Moreover, this research renamed the identified GRAS TFs in accordance with their chromosomal locations via the nomenclature by utilizing the GRAS + sequence number. Through employing the ProtParam (https://web.expasy.org/protparam/, accessed on 28 May 2024) online software, physicochemical properties were explored for their theoretical pI, amino acid number, molecular weight (MW), and instability coefficient (aliphatic index). Through utilizing DeepLoc-2.0 (https://services.healthtech.dtu.dk/service.php?DeepLoc-2.0, accessed on 28 May 2024), the subcellular location analysis of GRAS genes was completed [39].

### 2.3. Phylogenetic Relationship Analysis

The amino acid sequences of the GRAS protein found in *H. citrina* Baroni, *A. thaliana* and *O. sativa* (Tables S1 and S2) were processed using MEGA11.0 software for sequence alignment. Following this, phylogenetic trees were created utilizing the neighbor-joining (NJ) technique within MEGA11, based on 1000 bootstrap resampling iterations in MEGA11.0 software [36,40]. These trees were visualized using the Evolview v3 (http://www.evolgenius.info/evolview/#/treeview, accessed on 14 June 2024) online software [41].

### 2.4. Analysis of Gene Structure, Structural Domains, and Conserved Motifs

The genome annotation file of daylily, formatted in GFF3, was taken as the basis for choosing TBtools software to explore all genes’ structures [36]. By using the NCBI CD-search online tool (https://www.ncbi.nlm.nih.gov/Structure/bwrpsb/bwrpsb.cgi, accessed on 28 May 2024) with default parameters, predictions were then conducted on their structural domains [38]. The conserved motifs of GRAS proteins were explored through utilizing the MEME (http://meme-suite.org/, accessed on 29 May 2024) online software [42], which indicated differences between the HcGRAS family members. The parameters for the conserved motifs were optimized as follows: the number of motifs was set to a maximum of 20, and the optimal width of residues was between 6 and 200. These findings mentioned before were ultimately visualized through TBtools [36].

### 2.5. Analysis of cis-Acting Elements

After obtained gene IDs were identified, TBtools software (v2.310) facilitated the extraction of 2000 bp upstream nucleotide sequences for each gene from the GFF3 file. Subsequently, the promoter regions were analyzed using the online tool PlantCARE (https://bioinformatics.psb.ugent.be/webtools/plantcare/html/, accessed on 5 June 2024) in order to identify the elements that act as cis-regulatory sequences in the promoter region [43].

### 2.6. Chromosomal Distribution and Gene Duplication

The locations of *HcGRAS* genes within the genome were assessed visually using TBtools software, with the data sourced from the daylily genome [36]. To explore the gene duplication events of the GRAS genes, default parameters were used to adopt the Multiple Collinear Scanning Toolkits (MCScanX) [44]. The homology of *GRAS* genes between *H. citrina* Baroni and seven other plants (*Z. mays*, *B. distachyon*, *A. thaliana*, *S. italica*, *S. bicolor*, *O. sativa* and *S. lycopersicum*) was explored through the dual synteny plotter project within TBtools software. Non-synonymous (Ka) and synonymous substitution sites (Ks) of all of the duplicated GRAS genes were calculated through the use of the Simple Ka/Ks Calculator module of TBtools.

### 2.7. Gene Ontology Function of GRAS Genes and Kyoto Encyclopedia of Genes and Genomes Pathway Enrichment Analysis

Function annotations for the protein sequences of 78 *HcGRAS* TF family genes were performed based on the eggNOG-MAPPER database (http://eggnog-mapper.embl.de/, accessed on 5 June 2024) [45]. TBtools software was leveraged to conduct the Gene Ontology (GO) function of the *HcGRAS* gene and Kyoto Encyclopedia of Genes and Genomes (KEGG) pathway enrichment analysis on the *HcGRAS* genes. Furthermore, the online tool ChiPlot (https://www.chiplot.online/, accessed on 18 June 2024) was also used for mapping and visualization.

### 2.8. Protein–Protein Interaction (PPI) Network Analysis and Subcellular Localization

The interaction data of *O. sativa* were first downloaded from the STRING protein–protein interaction database (PPD) (http://string-db.org/, accessed on 20 June 2024) [46]. Subsequently, the representative transcriptional protein sequences were extracted from the genome of daylily using TBtools. Moreover, to obtain the protein interaction relationship in dayliy, the abovementioned obtained files were dragged into the TBtools PPI Predict module [36]. After screening, a network interaction map was established for the daylily’s GRAS proteins. Next, Cytoscape software (v3.9.1) was leveraged to visualize and analyze the interaction [47].

The coding sequence of HcGRAS38, excluding the termination codon, was cloned into the vector pGreenII 62-SK-GFP, resulting in the creation of pGreenII 62-SK-HcGRAS38-GFP using Not I and Spe I enzymes (TaKaRa, Dalian, China). The co-infiltration of the appropriate vectors into the leaves of *Nicotiana benthamiana* (40 d old) was facilitated by the strain GV3101. The GFP fluorescence signal was examined using confocal fluorescence microscopy (Leica TCS SP8, Wetzlar, Germany) 48 h following the transient transformation conducted in darkness.

### 2.9. Analysis of Tissue Expression Characteristics and RT–PCR

The gene expression data of daylily were collected from the NCBI (https://www.ncbi.nlm.nih.gov, accessed on 20 May 2024). Transcriptome changes in daylily at various flower development stages (SRP243199) were measured through performing the RNA-seq experiment [48]. In this experiment, the RNA samples were divided into six stages according to their sizes, which were stage A (2.1–2.5 cm), stage B (4.1–4.5 cm), stage C (5.1–5.5 cm), stage D (7.1–8.0 cm), stage E (full opening) and stage F (flower senescence) [48]. Transcriptome changes in daylily by spraying exogenous JA were measured through performing the RNA-seq experiment [49]. When the seedlings grew to the seven-leaf stage in spring, we selected disease-free seedlings with roughly the same growth conditions and sprayed 1 mg/L exogenous JA evenly on each plant with about 200 mL until the solution drips from the leaves. Then, we covered the entire plant with a transparent plastic bag for 8 h to prevent the treatment solution from evaporating. The fifth to seventh leaves (new leaves) of each daylily seedling were selected for each treatment at 1, 3, 5, 7, and 9 days after spraying (named JA-1d, JA-3d, JA-5d, JA-7d, JA-1d, and JA-9d), and the control check (CK) was sprayed with equal amounts of 0 mmol/L JA prepared by adding equal amounts of anhydrous ethanol and distilled water, with the samples designated ck-1d, ck-3d, ck-5d, ck-7d, and ck-9d (PRJNA1094559 and PRJNA1097583). Transcriptome changes in the 5-leaf stage daylily by spraying 2 mgL^−1^ SA were measured through performing the RNA-seq experiment (PRJNA1056505) [50]. This transcriptomic information facilitated the assessment of the differential expression of *GRAS* genes after 0, 3, 6, 8, and 12 d to investigate the response mechanism of *H. citrina* (we selected healthy plants of daylily that had been replanted for two weeks) to rust infection (PRJNA891118) [51]. The transcriptome data of daylily exposed to *Thrips palmi* (*T. palmi*) feeding stress were also measured. *T. palmi* was starved for 12 h prior to the test to ensure adequate feeding induction on plants. On the day of the experiment, *T. palmi* was transported to the net room and released to the seven-leaf stage daylily such that there were ~90 individuals per plant. Daylily in this treatment group was individually covered with a 60-mesh insect-proof net to prevent *T. palmi* from escaping. Plant leaves were collected at 1, 3, 5, 7, and 9 days after the introduction of *T. palmi* (named thirps-1d, thirps-3d, thirps-5d, thirps-7d, and thirps-9d), and the leaves harvested concurrently from the CK group were utilized as controls (designated ck-1d, ck-3d, ck-5d, ck-7d, and ck-9d 5) (PRJNA1094559) [52]. The *GRAS* genes were filtered to determine their potential functions in flower development. The expression was normalized via the “Normalized” scaling approach in TBtools, and this research also employed TBtools to generate heatmaps in accordance with the transformed data of log2 (FPKM + 1) values [36].

The RNA was isolated from various parts of the daylily using a kit specifically designed for RNA extraction (Tiangen Biotech, Beijing, China). First-strand cDNA was synthesized using a PrimeScriptTM RT reagent kit (TaKaRa, Dalian, China). PCR was conducted utilizing SYBR^®^ *Premix Ex Taq*^TM^ II (TaKaRa, Dalian, China) with gene-specific primer sets on the ABI QuantStudio (TM) 6 Flex System (Applied Biosystems, Foster City, CA, USA). The gene expression levels were quantified through an analysis of three separate biological samples, along with an assessment of three technical replicates, to determine the relative expression levels using the comparative Ct (2^−ΔΔCt^) [53] method, with *Actin* (used by [54]) serving as a control (Table S3). An analysis of statistical significance and data was conducted using the GraphPad Prism6 (GraphPad Software, Inc., San Diego, CA, USA). The data are presented in the form of “mean ± SD”. The least significant difference test (LSD) was utilized to determine the comparison between various samples, and various letters exhibit significant differences at the level of *p* < 0.05.

## 3. Results

### 3.1. Identification of GRAS Genes in Daylily

The *GRAS* members were renamed *HcGRAS1* to *HcGRAS78* (Table S2) according to the chromosomal location. A systematic analysis was conducted on basic characteristics, such as subcellular localization, the isoelectric point, the protein molecular weight, and the gene coding sequence. Among the 78 *HcGRAS* proteins, *HcGRAS71* was the smallest with 199 amino acids and *HcGRAS30* was the largest with 771 amino acids. In these proteins, the range of the molecular weight was from 23.21 kDa (*HcGRAS71*) to 84.23 kDa (*HcGRAS30*). The range of the isoelectric point was from 4.74 (*HcGRAS75*) to 9.51 (*HcGRAS44*), in which the median was 5.54. The instability index was 32.33 to 64.18, the aliphatic index was 70.08 to 95.37, and the hydropathicity grand average was −0.549 to 0.08. The *GRAS* domain was included in each putative protein that was encoded by *HcGRAS* genes. In accordance with predicted subcellular localization, 68 *HcGRAS*s were located in the nucleus, and (*HcGRAS20* and *HcGRAS71*) and 8 (*HcGRAS3*, *HcGRAS13*, *HcGRAS51*, *HcGRAS57*, *HcGRAS58*, *HcGRAS61*, *HcGRAS70* and *HcGRAS77*) were located in the cytoplasm/nucleus (Table S2).

### 3.2. Phylogenetic Tree of HcGRASs

**Figure 1:**
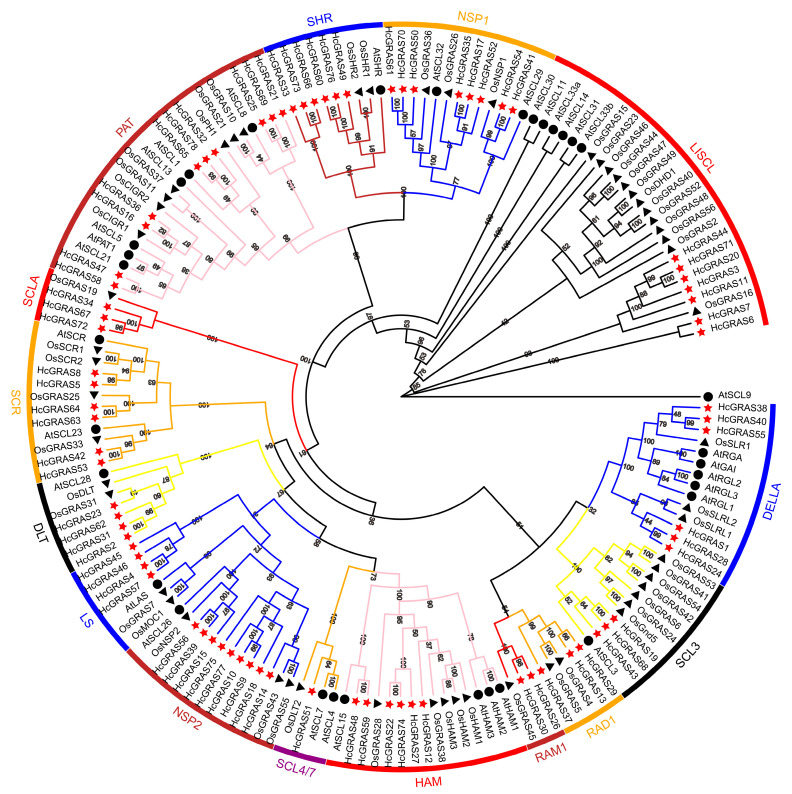
The phylogenetic tree of daylily, *Arabidopsis*, and rice GRAS proteins constructed by MEGA11.0. The tree shows 16 phylogenetic subfamilies marked with different colored fonts on a white background. The GRAS proteins derived from *A. thaliana* and *O. sativa* are prefixed ‘At’ and ‘Os’, respectively.

For the purpose of revealing the evolutionary relationship of *GRAS* genes between *A. thaliana*, *O. sativa* and *H. citrina*, this research set up a neighbor-joining phylogenetic tree in accordance with the 33 *AtGRAS*s from *A. thaliana*, 56 *OsGRAS*s (Table S1) from *O. sativa*, and 78 *HcGRAS*s (Table S2) from *H. citrina* Baroni (Figure 1). The results showed that 78 *HcGRAS*s were clustered into 15 subfamilies: DELLA, SCL3, RAD1, RAM1, HAM, SCL4/7, NSP2, LS, DLT, SCR, SCLA, PAT, SHR, NSP1, and LISCL. *HcGRAS1*, *HcGRAS24*, *HcGRAS28*, *HcGRAS38*, *HcGRAS40* and *HcGRAS55* are DELLA subfamily members. *HcGRAS2*, *HcGRAS23*, *HcGRAS31* and *HcGRAS62* are DLT subfamily members. *HcGRAS12*, *HcGRAS22*, *HcGRAS27*, *HcGRAS74*, *HcGRAS48* and *HcGRAS59* are HAM subfamily members. The LS subfamily has four members, including *HcGRAS4*, *HcGRAS45*, *HcGRAS46*and *HcGRAS57*. *HcGRAS3*, *HcGRAS6*, *HcGRAS7*, *HcGRAS11*, *HcGRAS20*, *HcGRAS44* and *HcGRAS71* are LISCL subfamily members. The NSP1 subfamily has eight members, including *HcGRAS41*, *HcGRAS54*, *HcGRAS17*, *HcGRAS35*, *HcGRAS50*, *HcGRAS52*, *HcGRAS61*, and *HcGRAS70*. *HcGRAS56*, *HcGRAS75*, *HcGRAS9*, *HcGRAS10*, *HcGRAS14*, *HcGRAS15*, *HcGRAS18*, *HcGRAS39* and *HcGRAS77* are members of the NSP2 subfamily. *HcGRAS34*, *HcGRAS67* and *HcGRAS72* are SCLA subfamily members. *HcGRAS13*, *HcGRAS29*, and *HcGRAS37* are RAD1 subfamily members. *HcGRAS16*, *HcGRAS21 HcGRAS25*, *HcGRAS69*, *HcGRAS32*, *HcGRAS36*, *HcGRAS47*, *HcGRAS58*, *HcGRAS65* and *HcGRAS78* are PAT subfamily members. *HcGRAS26* and *HcGRAS30* are RAM1 subfamily members. *HcGRAS19*, *HcGRAS43* and *HcGRAS68* are SCL3 subfamily members. *HcGRAS51* is an SCL4/7 subfamily member. *HcGRAS21*, *HcGRAS25* and *HcGRAS69* are SCL8 subfamily members. *HcGRAS5*, *HcGRAS8*, *HcGRAS42*, *HcGRAS53*, *HcGRAS63* and *HcGRAS64* are SCR subfamily members. *HcGRAS33*, *HcGRAS49*, *HcGRAS60*, *HcGRAS66*, *HcGRAS73* and *HcGRAS76* are SHR subfamily members.

### 3.3. Gene Structure, Domain, and Conserved Motif Composition Analysis of HcGRAS Genes

**Figure 2:**
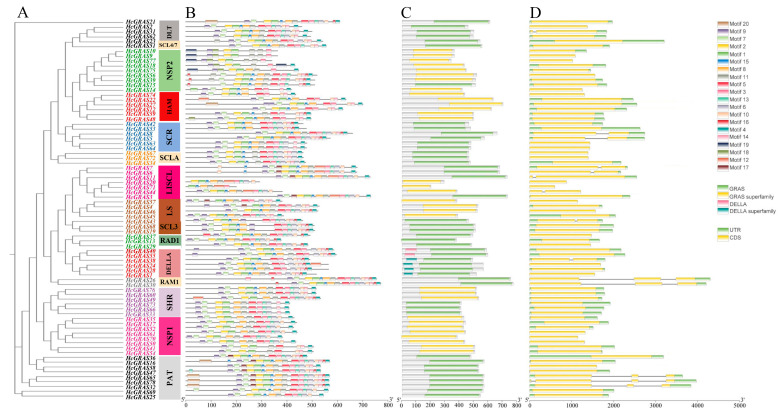
The analysis of phylogenetic relationships, gene structure, and distribution of motifs among *HcGRAS* genes. The phylogenetic tree was generated using the NJ method with 1000 replicates on each node (**A**). The conserved motif distribution of GRAS proteins (**B**); motifs 1–20 are represented by boxes of different colors. The conserved functional domains of *HcGRAS*s (**C**). Gene structures of *HcGRAS* genes (**D**).

Based on phylogenetic analysis, the identified 78 *HcGRAS* proteins were divided into 14 subfamilies (Figure 2A). Through leveraging the MEME online tool, an analysis of the *HcGRAS* protein sequences revealed the presence of 20 different conserved motifs (Figure 2B). Changes in different proteins were indicated by the number and distribution of these motifs and 20 different conserved motifs were found (Figure 2B and Figure S2). It is found that motifs 1, 2, 3, 9, and 4 found distribution within the *HcGRAS* family. A similar motif composition was often found in *HcGRAS* members of the same subfamily. The DELLA subfamily included motifs 9, 7, 17, 1, 15, 8, 11, 5, 3, 6, 10, 14 and 4; the SHR subfamily included motifs 9, 7, 2, 1, 8, 11, 5, 3, 13, 6, 14 and 4; and the PAT1 subfamily included motifs 9, 7, 2, 1, 15, 11, 5, 3, 10, 14, 16 and 4. Certain motifs were only distributed in the pattern’s specific locations. An example is that motif 9 (73, ~93.59%) and motif 7 (69, ~88.46%) were distributed at the pattern’s starting all the time and motif 4 (72, ~92.31%) at the pattern’s end. Conserved domain analysis showed that HcGRAS proteins consisted of *GRAS*, the *GRAS* superfamily, DELLA and the DELLA superfamily (Figure 2C), suggesting significant levels of preservation throughout their evolutionary history. Additionally, a structural map containing the CDS and UTR was constructed based on the genome sequence (Figure 2D), contributing to an understanding of the structural composition of *HcGRAS* genes. The coding sequences of 78 *HcGRAS*s are shown in Table S2. Gene structure analysis revealed that a similar gene structure was observed in members of the same evolutionary clade.

### 3.4. Chromosomal Localization, Collinearity and Evolutionary Analysis of HcGRAS Gene Members

**Figure 3:**
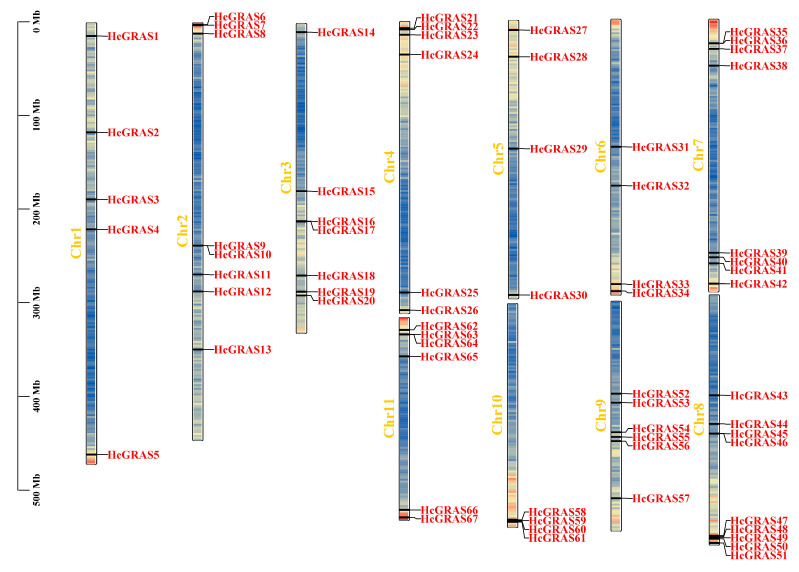
Chromosome distribution of *GRAS* genes on daylily (Chrs 1–11). Each *HcGRAS* gene named is exhibited on daylily Chrs, in which the Chr number is marked at each strip’s edge. The scale ranging from 0 to 500 Mb denotes the chromosome length, while the internal lines within the chromosomes reflect the density of the genes.

The results indicated that 67 *HcGRAS* members were not evenly distributed on 11 Chrs and 11 *HcGRAS* members were evenly distributed at unanchored points. Chr 8 had the most distribution with nine members, followed by Chrs 2 and 7 with eight members, Chr 3 with seven members, Chrs 4, 9 and 11 with six members, Chr 1 with five members, and Chrs 5, 6 and 10 with four members (Figure 3).

**Figure 4:**
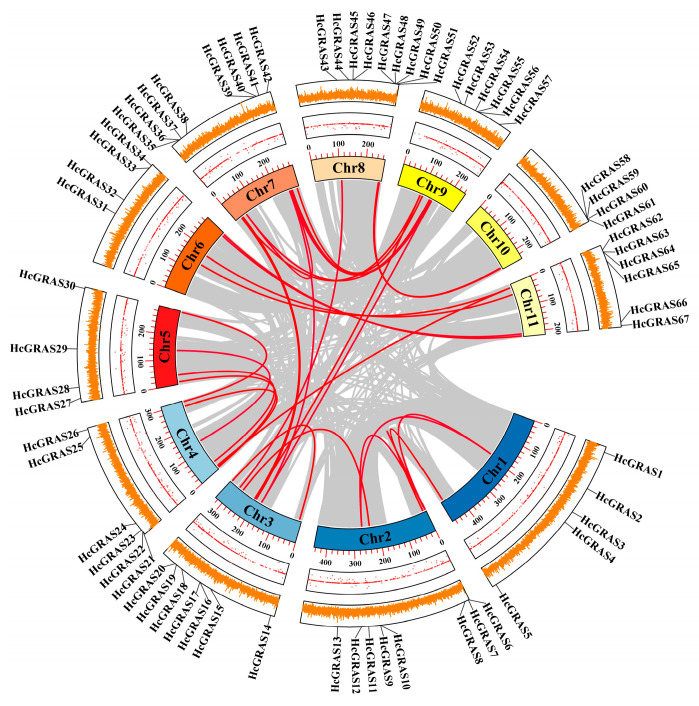
The chromosomal positions and the relationships among different chromosomes concerning *GRAS* family genes in *H. citrina* Baroni. Red lines indicate the events of duplication that have been identified.

**Figure 5:**
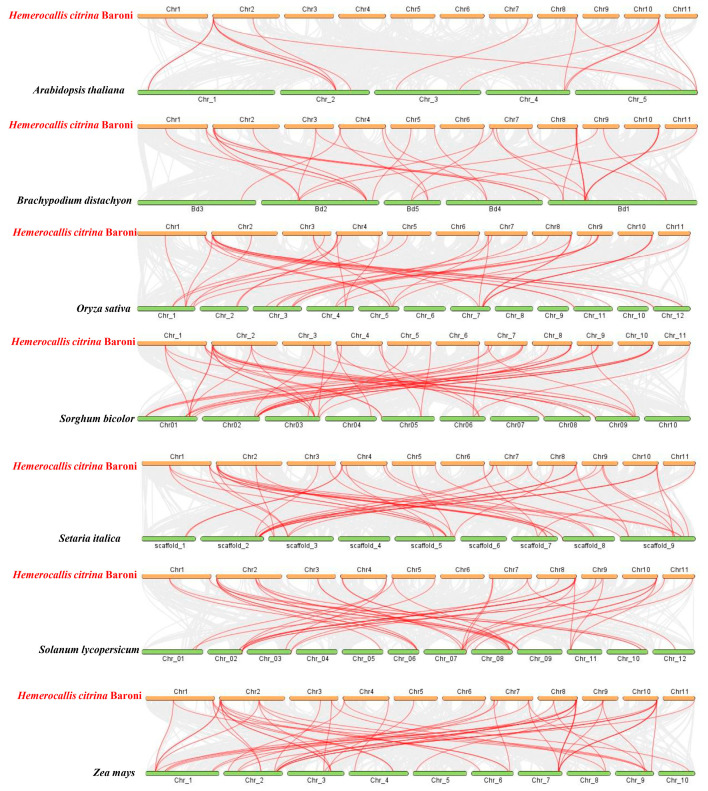
Collinearity analysis between the GRASs of *H. citrina*, *A. thaliana*, *B. distachyon*, *O. sativa*, *S. bicolor*, *S. italica*, *S. lycopersicum* and *Z. mays*.

The intra-species collinear analysis was carried out for the purpose of deciding if gene duplication resulted in the *HcGRAS* gene family’s evolution (Figure 4 and Figure 5). More pairs of duplicated segments were found within *HcGRAS*s, which suggested that segmental duplication played a significant role in genetic diversity. Then, intra-species syntenic maps of *H. citrina* Baroni that have seven species were constructed to identify orthologous genes using TBtools (Figure 5 and Table S4). A total of 44 *HcGRAS* genes exhibited collinear relationships with *A. thaliana* (7), *B. distachyon* (33), *Oryza sativa* (32), *S. bicolor* (33), *S. italica* (32), *S. lycopersicum* (31), and *Z. mays* (27). The orthologous gene pairs identified between daylily and the remaining seven species totaled, including *B. distachyon*, *S. bicolor*, *S. italica*, *Arabidopsis*, tomato, rice, and maize, were 15, 50, 6, 53, 52, and 55, respectively.

### 3.5. Identification and Distribution of cis-Regulatory Elements in the HcGRAS Promoter

**Figure 6:**
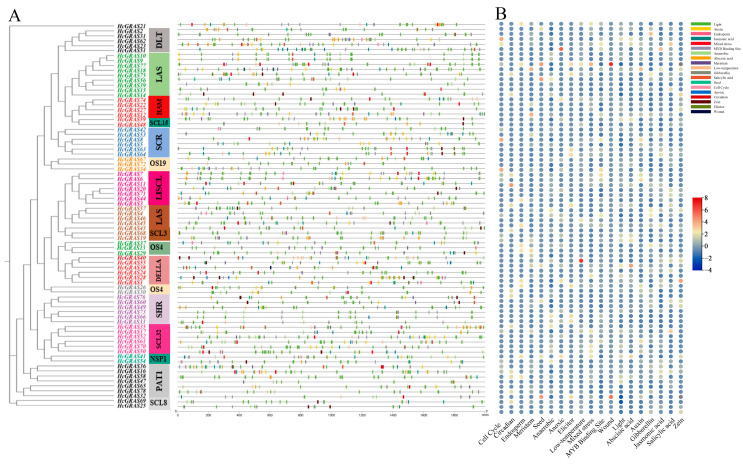
A prediction of transcription factor binding sites in the promoters of *HcGRAS* (**A**); boxes of different colors represent different transcription factor families. The number of functional elements in *HcGRAS*s (**B**).

Specifically, this research extracted a total of 2000 base pairs of the transcription start codon of *HcGRAS* genes in order to analyze their potential biological functions through the use of the PlantCARE database. Three major responsive elements in total were identified, such as plant growth and development, response to abiotic stress, and hormone response (Figure 6 and Table S5). Among these, 1 to 22 elements responsive to light were detected in 78 genes, which indicated that a significant portion of the *HcGRAS* genes is influenced by light signaling pathways. Each of the 78 genes included cis-acting elements influencing hormonal response and developmental processes. Elements related to hormones contained GA response, SA, JA, auxin, and abscisic acid (ABA). In contrast, elements related to development contained MYB binding site response, circadian control, anoxic specific inducibility, meristem expression, endosperm, cell cycle regulation, anaerobic induction, seed-specific regulation, and zein metabolism regulation. Furthermore, numerous *HcGRAS* genes exhibited cis-acting elements associated with abiotic stress factors, including responses to low temperatures, combined environmental stress, and wounds. As the promoter regions of 78 *HcGRAS*s contained various types of cis-elements, they may be involved in diverse regulatory pathways, environmental stresses, and biological processes.

### 3.6. Functional Annotation of HcGRAS TFs

**Figure 7:**
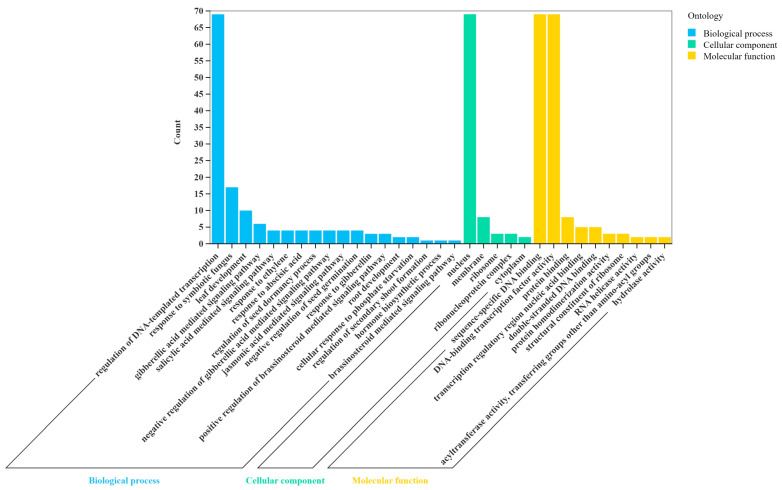
GO enrichment analysis of *HcGRAS* genes.

In order to further explore biological functions of the *HcGRAS* TF family, this research annotated 78 *HcGRAS* genes’ proteins (Figure 7 and Table S6). It was found that 69 *HcGRAS* genes were annotated and assigned to three broad categories, which were biological process (BP), cellular component (CC), and molecular function (MF). For the high enrichment of *HcGRAS* genes for organic cyclic compound binding and nucleic acid binding, the *HcGRAS* TF family is found to contribute much to affecting transcription regulation. Among cellular components, *HcGRAS* genes were enriched in the nucleus and membrane. In biological processes, mediated signaling pathways, responses to hormonal and environmental stimuli, growth and development processes, and biomass synthesis were enriched with more *HcGRAS* genes. Among them, growth and development processes, including dormancy processes, tissue development and plant organ development, environmental stimulus responses, including responses to symbiotic fungus and phosphate starvation responses, and hormonal stimulus responses, including SA, GA, ABA, brassinosteroid and JA signaling pathways, all indicate that the *HcGRAS* TF family is likely to adapt plants to different environments. Furthermore, KEGG enrichment analysis demonstrated that 32 out of the 78 identified *GRAS* genes were significantly associated with the specific signaling pathway related to plant hormones (Table S7).

### 3.7. Protein–Protein Interaction Analysis of the HcGRAS TF Family

**Figure 8:**
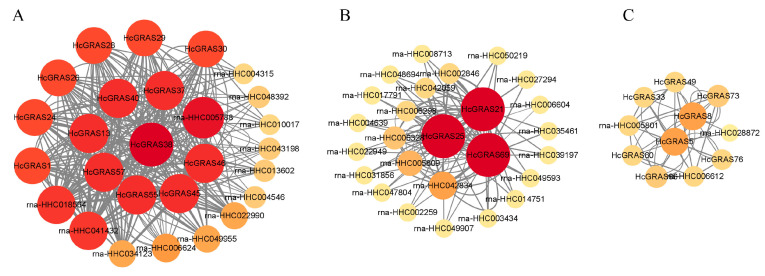
Diagram illustrating reciprocal interactions among proteins. Each circle represents a protein. PPI between DELLA subfamily members and other proteins (**A**). PPI between SCL8 subfamily members and other proteins (**B**). PPI between SCR subfamily members and SHR subfamily members (**C**).

An analysis of the interaction network among 78 HcGRAS proteins indicated that 25 of these genes established interactions, while interaction relationships were not formed in 53 genes (Figure 8). Figure 8A implies that those proteins are crucial and the DELLA subfamily members HcGRAS1, HcGRAS24, HcGRAS28, HcGRAS38, HcGRAS40 and HcGRAS55, which is a family of proteins that regulate an adverse environment by interacting with GA. The other proteins, all of which belong to the SCR, OS4, LS, and SHR subfamilies, interact with them, particularly the HcGRAS38 protein, serving as the core connecting DELLA proteins, which indicates that its role is necessary for the DELLA subfamily. HcGRAS21, HcGRAS25 and HcGRAS69, which belong to the SCL8 subfamily, are the key factors of protein interaction in Figure 8B, and they can interact with many proteins in daylily. In Figure 8C, HcGRAS5 and HcGRAS8, which belong to the SCR subfamily, play an important role in the PPI network. Meanwhile, HcGRAS33, HcGRAS49, HcGRAS60, HcGRAS66, HcGRAS73, and HcGRAS76, which belong to the SHR subfamily, suggest that there is a protein interaction relationship between SHR and SCR subfamilies.

### 3.8. Subcellular Localization of HcGRAS18 Protein

**Figure 9:**
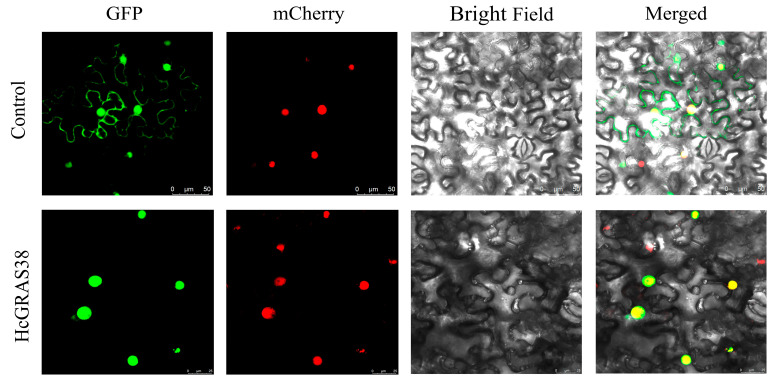
A fusion protein of HcGRAS38 with GFP was analyzed for its subcellular distribution in the leaves of *N. benthamiana*.

In order to confirm the expected subcellular localization of HcGRAS38, daylily HcGRAS38-GFP was co-expressed with the nucleus marker (mCherry) vector, subsequently transferring the recombinant plasmid into *Agrobacterium tumefaciens*, and we injected it into *N. benthamiana*. After a period of 48 h, observations were made with a confocal laser scanning microscope, revealing that HcGRAS38 was localized to the nucleus (Figure 9).

### 3.9. Expression Analysis of HcGRAS Genes

**Figure 10:**
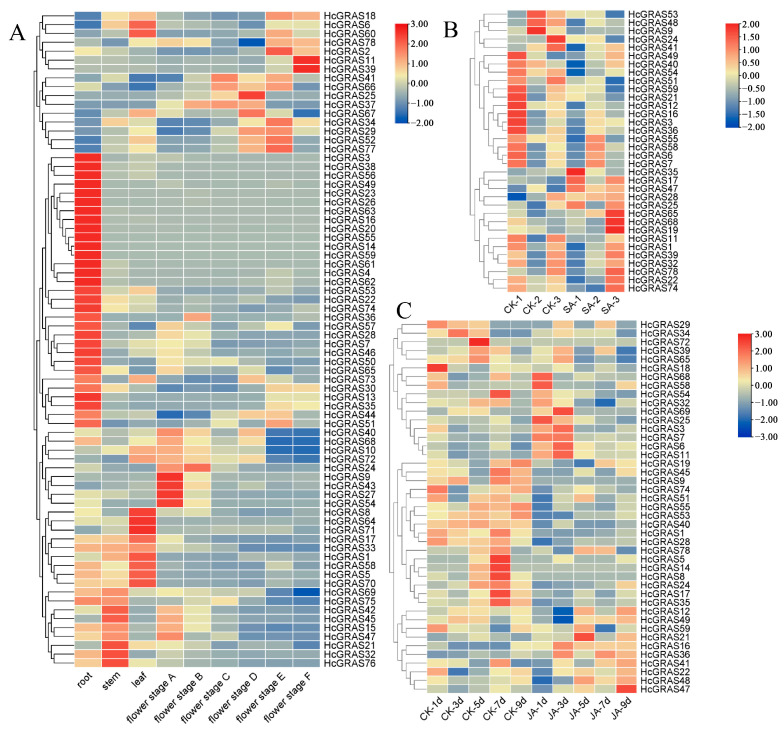
Analysis of expression patterns in *HcGRAS*s. (**A**) Heatmap illustrating the expression profiles of 78 *HcGRAS* genes at various stages of floral development. (**B**) Expression patterns of *HcGRAS*s under salicylic acid treatment. (**C**) Expression patterns of *HcGRAS*s under jasmonic acid treatment. Different colored boxes exhibit row-scaled FPKM values, with expression gradually increasing from blue to yellow to red at a time.

Transcriptome data from different developmental stages of daylily were analyzed to understand *HcGRAS* genes’ expression patterns (Figure 10A). Certain *HcGRAS* genes exhibited a consistent pattern of high expression at various flower development stages, including *HcGRAS21*, *HcGRAS2*, *HcGRAS77*, *HcGRAS18*, *HcGRAS6*, and *HcGRAS1*. Each OS19 subfamily member exhibited high expression at different stages, but there were great differences in the number of transcripts from members of other subfamilies. An example is that the SCL32 subfamily’s *HcGRAS61* almost cannot be detected in transcripts, with the overexpression of other members. The SCL8 subfamily’s *HcGRAS25* was highly expressed at the flower stages A to D.

To investigate the expression patterns of *HcGRAS* genes under hormone stress, a heatmap of the expression profiles was performed for their predicted cis-acting elements associated with SA and JA. The transcriptomic analysis of daylily exposed to SA was conducted to elucidate the expression patterns of *HcGRAS* genes (Figure 10B). After SA treatment, the level of expression of some genes increased significantly including *HcGRAS35*, *HcGRAS17*, *HcGRAS47*, and *HcGRAS28*. Some other genes showed decreased expression levels after the SA treatment of *HcGRAS53*, *HcGRAS48*, and *HcGRAS9*. The gene expression heatmap results after JA treatment was shown in Figure 10C. Notably, *HcGRAS16*, *HcGRAS36*, *HcGRAS11*, *HcGRAS6*, and *HcGRAS7* showed high expression under JA treatment. Additionally, *HcGRAS55*, *HcGRAS53*, *HcGRAS40*, *HcGRAS1*, and *HcGRAS28* were down-regulated. These observations underscore the significant functions of *HcGRAS* genes in regulating plant development and their responses to stressors mediated through SA and JA.

**Figure 11:**
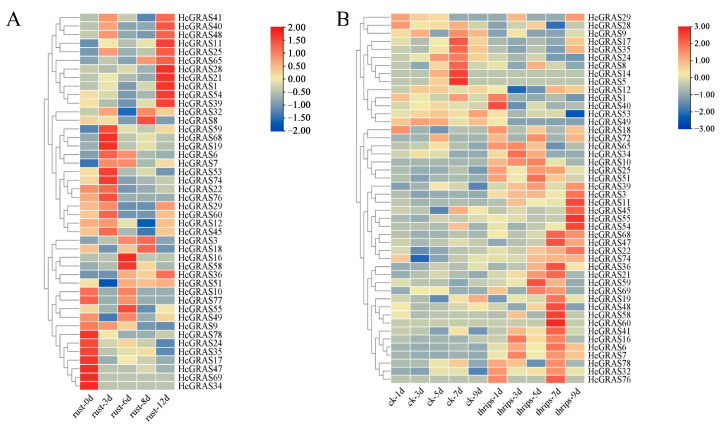
Analysis of expression patterns in *HcGRAS*s under biotic stress. (**A**) Expression patterns of *HcGRAS*s after rust infection. (**B**) Expression patterns of *HcGRAS*s exposed to *Thrips palmi* feeding stress. Different colored boxes exhibit row-scaled FPKM values, with expression gradually increasing from blue to yellow to red at a time.

An investigation was conducted to assess the involvement of HcGRAS genes when subjected to biotic stress. The expression of *HcGRAS* genes was assessed following stress from *Puccinia hemerocallidis* infection and feeding by *T. palmi*. The expression levels of *HcGRAS11* and *HcGRAS65* gradually increased after rust infection, and the expression level of *HcGRAS9* gradually decreased (Figure 11A). For example, genes such as *HcGRAS21*, *HcGRAS25*, *HcGRAS40*, *HcGRAS41*, and *HcGRAS48* have the highest expression levels after 12d of treatment, while genes such as *HcGRAS17*, *HcGRAS24*, *HcGRAS34*, *HcGRAS35*, and *HcGRAS69* show the highest levels of expression at 0 d and then decrease. Some genes, such as *HcGRAS16*, *HcGRAS19*, *HcGRAS55*, *HcGRAS58*, and *HcGRAS59*, showed higher expression levels at 3d and 6d after rust infection. After thrips stress treatment, the expression levels of *HcGRAS5*, *HcGRAS9*, *HcGRAS14*, *HcGRAS24*, *HcGRAS49* and *HcGRAS53* were higher in the control group (Figure 11B). The expression levels of *HcGRAS3*, *HcGRAS10*, *HcGRAS11*, *HcGRAS*25, *HcGRAS32*, *HcGRAS39*, *HcGRAS51* and *HcGRAS76* were relatively high in the treatment group (Figure 11B).

**Figure 12:**
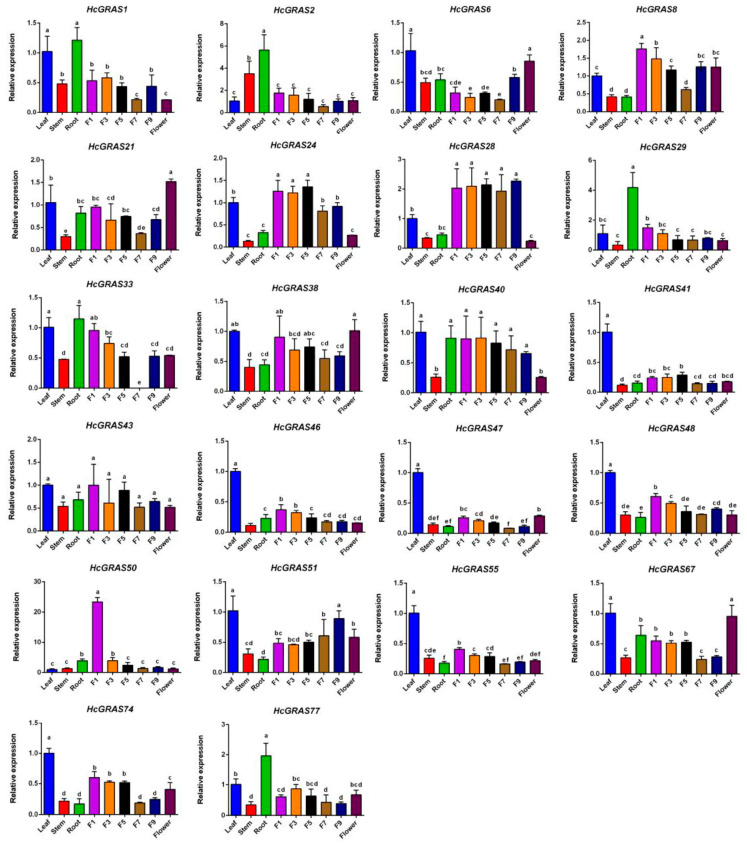
Expression analysis of *HcGRAS*s in different tissues. Different lowercase letters indicate significant differences by LSD (*p* < 0.05).

The expression levels of 22 members in four organs (flower, root, stem, and leaf) and five flower stages were detected using real-time PCR (Figure 12) to explore the physiological functions of *GRAS* genes within daylily. There was the highest expression in the leaf for seven genes (*HcGRAS41*, *HcGRAS46*, *HcGRAS47*, *HcGRAS48*, *HcGRAS51*, *HcGRAS55* and *HcGRAS74*) and in the root for five genes (*HcGRAS1*, *HcGRAS2*, *HcGRAS29*, *HcGRAS33* and *HcGRAS77*). Significant differences were identified in the expression levels of *HcGRAS* genes at various flower development stages. Compared to the full-open flower stage, there was higher gene expression before the early flower stage. Nine genes (*HcGRAS8*, *HcGRAS21*, *HcGRAS29*, *HcGRAS33*, *HcGRAS40*, *HcGRAS43*, *HcGRAS48*, *HcGRAS50* and *HcGRAS55*) exhibited the highest expression at the F1 stage (including F1, F3, F5, F7, F9 and flower), while three genes (*HcGRAS6*, *HcGRAS38*, and *HcGRAS67*) exhibited the highest expression at the full-open flower stage (flower).

## 4. Discussion

### 4.1. HcGRAS Gene Structure and Evolutionary Analysis

The identification of gene families has been conducted in an increasing number of species with the progress of genome sequencing technology. The molecular mechanisms of plant *GRAS* TFs for addressing environmental stress have been determined after the identification and functional analysis of *GRAS* TFs in multiple crops and cash crops. However, no relevant studies have been conducted in daylily. In this study, the bioinformatics method was used to identify 78 *GRAS* TFs from the daylily genome. The number of *GRAS* genes in daylily was higher compared to that within *A. thaliana* (34) and rice (57) [2] and was close to that in apple (78) [8] and *M. acuminata* var. *DH-Pahang* (73) [11]. There were differences between 78 *HcGRAS* genes in the theoretical isoelectric point, molecular weight and nucleotide sequence length, suggesting the complexity of the daylily genome and the functional difference in HcGRAS genes in various microenvironments.

This research set up a phylogenetic tree by selecting *A. thaliana AtGRAS*, rice *OsGRAS* and daylily *HcGRAS* to explore the evolutionary relationships of *GRAS* TFs in different species. The results of *A. thaliana* and rice allowed for the classification of the *HcGRAS* subfamily, indicating that *GRAS* TFs were classified into 15 subfamilies: DELLA, SCL3, RAD1, RAM1, HAM, SCL4/7, NSP2, LS, DLT, SCR, SCLA, PAT, SHR, NSP1, and LISCL (Figure 1). Therefore, it is speculated that these subfamilies may be present in most monocotyledonous plants and have fundamental physiological functions that are conserved throughout evolution. In each *Arabidopsis* subfamily, the identification of at least one *HcGRAS* protein suggests that these *GRAS* subfamilies may play certain basic biological functions and are not lost during long-term evolution [2]. This study supports the hypothesis that separating the GRAS family is likely to precede the separation of daylily and *A. thaliana* [2,31]. Thus, further investigation is required for unique physiological functions of HcGRAS proteins. Among them, LS has the highest number of members (13, 16.67%), while SCL4/7 (HcGRAS51) has the lowest number of members. Similarly to the GRAS subfamilies found in other species, like *A. thaliana* [2], rice [18], soybean [10], millet [31], sugar beet [11], and *S. bicolor* [32], different subfamilies are likely to possess various differentiation capabilities throughout the course of evolution. The variations observed in these genes may hold considerable importance for the distinct roles they play across different species. However, limited research indicates that the mechanisms of adapting to environmental factors are linked to variations in differentiation among the subfamilies.

IDRs (intrinsically disordered regions) are constituted by the highly variable N-terminus of *GRAS* proteins, which include molecular recognition features and gene fragments that are easily exchanged. Through ordered and disordered transformations, the specific binding objects can be recognized in these functional regions [19,55]. Most *HcGRAS* genes have five major conserved domains: SAW, PFYRE, VHIID, LHR I, and LHR II (Figure 2B). The characteristic sequences of Asp, Ile, His and Val are included in the core VHIID domain. The HcGRAS proteins possess a region near their N terminus that is notably disordered, yet there are observed similarities across various subfamilies. A case in point is the involvement of the DELLA domain by the proteins of the DELLA subfamily at the N terminus (Figure 2C), which contributes to the functional diversity observed in GRAS proteins. In these regions, some residues are found to be conserved in various subfamilies, which may be necessary for the functions of *GRAS* proteins in various subfamilies. For instance, within woodland strawberry, alterations in the SAW domain have led to phenotypic variations [56].

The survey on the exons of *HcGRAS* genes shows that 1–4 exons are contained in each gene (Figure 2D). About 75.6% of *HcGRAS* genes have no intron, which is seen to be higher compared to those in millet (64.9%) [57] and sorghum (66.7%) [58] but lower than those in buckwheat (87%) [59]. Some subfamily members have a compact gene structure because SHR, LS, and SCL15 have only one intron or do not contain any introns. Also, genes with no intron are found in other families, such as the SAUR family [60], and DEAD box RNA helicase [61]. Genes that do not have an intron usually possess lower expression levels within plants [62]. Nonetheless, *GRAS* genes may directly originate from prokaryotes by means of horizontal gene transfer and duplication events [63,64]. Hence, the gene structure may be compact in the majority of *GRAS* members [65]. Genes with no intron continue to encode proteins in the transcription and translation process, which enables them to quickly respond to various environments [58,66]. Additionally, the expression of genes might not be influenced by the quantity of introns present within those genes [67].

The increase and development of the *GRAS* gene family can be attributed to the process of gene duplication, which serves as a driving evolutionary mechanism [68]. This research shows the uneven distribution of *HcGRAS* genes on 11 chromosomes, the majority of which were on chromosome 8. This phenomenon is potentially attributed to the localization characteristics of gene replication events, the structural and functional heterogeneity of chromosomes, and the disparities in evolutionary selection pressures [69,70]. Interestingly, there was no tandem duplication of *GRAS* genes on the chromosome of daylily. The majority of *GRAS* genes originated from segmental duplications, suggesting that this form of duplication serves as a primary mechanism for the expansion of gene families, which aligns with findings for *Benincasa hispida* [71]. Furthermore, the covariance of daylily with five monocotyledonous plants and two dicotyledonous plants was compared in this study. The daylily with a monocotyledon showed superior covariance compared to that with a dicotyledon. Compared to the interspecific collinearity, there is weaker intraspecific collinearity of *GRAS* genes in *Arabidopsis* and daylily, which indicates that gene replication is not the source of *GRAS* genes in various species (Figure 5) [72].

The Ka/Ks ratio is considered a crucial standard for measuring gene selection pressure [73]. Prior studies have shown that powerful purifying selection stress is experienced by *HcGRAS* genes involved in sea buckthorn duplication [74]. As shown in Table S8, all the *HcGRAS* genes are subject to purifying selection. In each gene, the Ka/Ks value is below 1, which indciates the purification state of each gene in the peach, possibly facilitating the elimination of harmful mutations and the maintenance of gene functions [75].

### 4.2. Predicting the Potential Functions of HcGRAS Genes

Cis-acting elements, such as enhancers and silencers, are crucial for the regulation of gene transcription in adjacent regions by engaging with trans-acting factors, which in turn affects the patterns and levels of gene expression [76]. The primary cis-acting components found within the initiation region of the *HcGRAS* TF family include elements responsive to hormones, those influencing light responses, and ones that react to environmental stress (Figure 6). There is the existence of many cis-acting hormones, including auxin, GA, SA and ABA. For gene promoters, except for a few genes, 80% involve GA response elements. The SCL32 and DLT subfamilies have witnessed a relatively good distribution of ABA response elements. Prior studies indicate that the structure of the N-terminal DELLA protein, belonging to the *GRAS* family of transcription factors, is pivotal in controlling various aspects of plant growth and development, as it functions within GA signaling pathways [77]. Although ABA can often promote stem elongation, seed germination and flower development, decreased cellular activity is observed in plants deficient in ABA and increased factor activity concerning ABA synthesis is also observed under adversity stress [78]. High salt stress activates ABA signaling, for example, leading to an accumulation of DELLA proteins, which inhibit plant growth and increase resistance [79]. Auxin has the functions of regulating stem growth rates, inhibiting lateral buds and promoting rooting, and some studies have found that ethylene and auxin interact with DELLA to regulate plant growth [80,81]. This implies that *GRAS* TFs in daylily can contribute much to regulating plant growth and development in the case of negative stress conditions as well.

The GO enrichment analysis revealed a significant number of genes that are responsive to various hormonal signals, such as pathways associated with SA, ABA, and JA, various environmental stimuli, such as reactions to cold temperatures, increased salinity levels, adaptations to other stress factors and processes related to growth and development, including the formation of plant organs, tissue differentiation, and dormancy. This is consistent with the findings observed in sugar beet [11]. By contrast, KEGG enrichment analysis just included a phytohormone signaling pathway, which suggests that *HcGRAS* transcription factors influence the regulation of plant growth and development by modulating hormone expression levels to adapt to challenging conditions. All of these findings further aligned with the analysis of the PPI network.

### 4.3. Gene Expression Profiles and RT-PCR Unveiled the Potential Roles of GRAS Genes in Flower Development, Hormone Response and Biotic Stress

It is reported that *GRAS* transcription factors are essential to flower development [31,32]. For *Arabidopsis*, a pentuple (*ga1–3 gai-t6 rga-t2 rgl1–1 rgl2–1*) DELLA mutant flowered earlier, which suggested that these transcription factors represent flowering repressors [82]. DELLA proteins function through repressing *FT* and *SPL3* included in regulating flowering onset, since early flowering mutant displays an increase in *FLOWERING LOCUS T* (*FT*) and *SQUAMOSA PROMOTER BINDING PROTEIN-LIKE 3* (*SPL3*) expression [82]. In tomatoes, a significant change occurred in the expression of genes included within pollen development and hormonal signaling for the flower transcriptome at anthesis [33]. A GA-constitutive response phenotype with increasing flowers was exhibited in the *procera* (*pro*) tomato mutant (*SlDELLA*) [83]. In grapes, there was an up-regulation of *VviRGA3* and *VviRGA5* in the process of fruit set and they were likely to regulate the transition from inflorescence to flower [84]. In this study, *HcGRAS1* was highly expressed in each stage, and *HcGRAS40* was expressed during the first four flower development stages, with low expression levels in the last two stages. *HcGRAS24* exhibited low expression levels during the first two stages and was downregulated in the subsequent four stages. Meanwhile, *HcGRAS55* was not detected or had low expression levels. This aligned with prior studies, indicating that the DELLA subfamily of *GRAS* genes plays a regulatory role in flower development.

Both DELLA and other subfamilies are included during the flower development process. In apples, there was significant modulation of the expression of *MdGRAS6* (LS), *MdGRAS122* (DELLA), *MdGRAS44* (PAT1), *MdGRAS53*, *MdGRAS107*, *MdGRAS26* (SHR), and *MdGRAS64* (LISCL) in the bud, which implies that they may contribute much to flowering. In the flower induction under hormonal treatment, through an observation of expression patterns of PAT1, LS, SCL, LISCL, SHR, and DELLA members, they were identified to be included in 6-benzyladenine, gibberellic acid and/or sugar-mediated flowering in apple trees as well [85]. It was also found the *LAM* gene had expression in all the floral meristematic tissues and young floral organs in strawberries [29]. These findings also showed *HcGRAS33* (SHR) and *HcGRAS74* (HAM) expressed at all stages of flower development, but the expression level showed a decreasing trend (Figure 10 and Figure 12). All these results indicate that daylily *GRAS* genes are likely to be included in flower development. Based on SA and JA treatment, as well as thrips disease and rust treatment, we have identified some specific expressed genes (Figure 10 and Figure 11), such as *HcGRAS25* (PAT) and *HcGRAS1* (DELLA) which provide some basis for future applications. The altered expression of *HcGRAS25* (PAT) aligns with trends observed in other species [25], indicating that the activation of PAT subfamily genes facilitates physiological adjustments in daylily, enabling adaptation to stress conditions and the maintenance of normal growth and metabolic activities.

## 5. Conclusions

This study focuses on identifying and characterizing the *GRAS* gene family within the daylily genome, using bioinformatics approaches to predict its structural and functional roles. The quality of the data could be enhanced as the majority were sourced from publicly available databases and the existing daylily data repository lacks completeness. A total of 78 *HcGRAS* transcription factors were identified, exhibiting an uneven distribution across 11 chromosomes. Transcriptional expression profiling, coupled with RT-PCR validation, confirmed the involvement of numerous *GRAS* genes in flower organ development and their response to SA and JA and resistance against rust and thrips pests. These findings provide a foundation for future research delving into the biological role of *HcGRAS* and offer a theoretical framework for its application in molecular breeding. Ultimately, this study contributes to the development of more efficient and precise breeding strategies for daylily, which may have broader implications for global agricultural advancements.

## Data Availability

All of the datasets supporting the results of this article are included within the article and its additional files. RNA-seq data from various tissue parts and treatments of daylily (accessions: SRX7583022, SRX7583021, SRX7583020, SRX7583019, SRX7583018, SRX7583017, SRX7583016, SRX7583015, SRX7583014, SRX24114404, SRX24114427, SRX24114432, SRX24114433, SRX24114408, SRX24114411, SRX24114415, SRX24114420, SRX24114423, SRX24114426, SRX24187491, SRX24187493, SRX24187496, SRX24187486, SRX24187490, SRX17912432, SRX17912437, SRX17912456, SRX17912459, SRX17912460, SRX23017183, SRX23017182, SRX23017181, SRX23017180, SRX23017179, SRX23017178) were systematically retrieved from the NCBI’s Sequence Read Archive (SRA) database.

## Supplementary Materials

The following supporting information can be downloaded at https://www.mdpi.com/article/10.3390/biology14070770/s1, Figure S1: Morphology of different tissue samples of daylily; Figure S2: Conserved sequence logo of GRAS proteins in daylily; Table S1: List of AtGRAS and OsGRASs used in this study; Table S2: List of 78 *Hemerocallis citrina* Baroni *GRAS* genes identified in this study; Table S3: Primer information for quantitative real-time PCR; Table S4: One-to-one orthologous relationships between *Hemerocallis citrina* Baroni and seven species; Table S5: Cis-regulatory elements in the promoter region of *HcGRAS* genes; Table S6: The GO enrichment analysis of the *GRAS* genes; Table S7: The KEGG pathway enrichment analysis of the *GRAS* genes; Table S8: Ka/Ks ratio distribution of gene pairs.
