# Supplementary figures and images for: Genome-Wide Identification of GRAS Gene Family in Daylily (Hemerocallis citrina Baroni) and Its Expression Profiles in Development, Hormone and Biotic Stress Response
Source: Biology (Basel). 2025 Jun 26;14(7):770. doi: 10.3390/biology14070770 (PMC12292224; doi:10.3390/biology14070770)

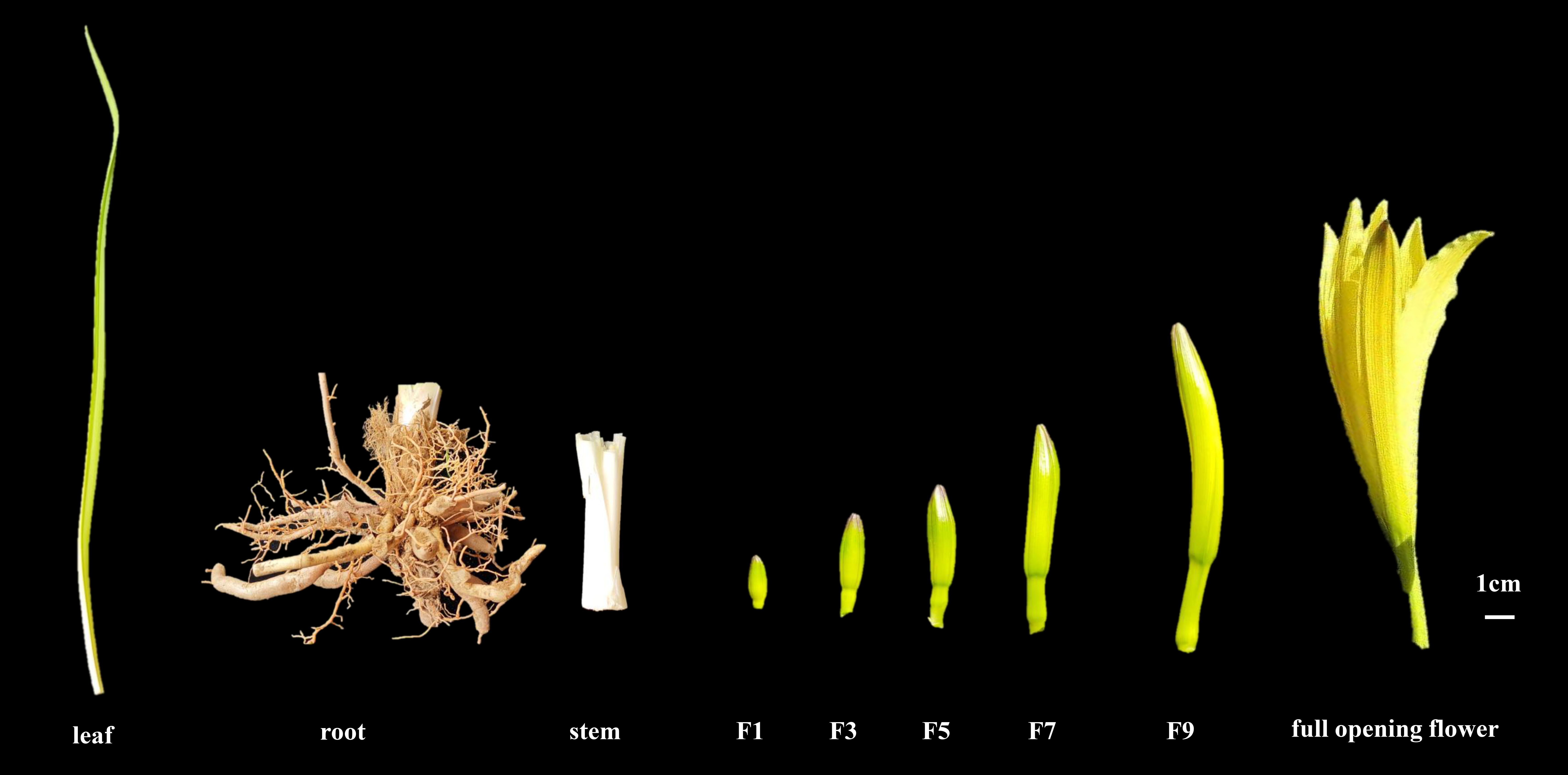

Supplement: Supplementary file 1 [file biology-14-00770-s001.zip › Figure S1.jpg]
